# Exploitation of novel wild type solventogenic strains for butanol production

**DOI:** 10.1186/s13068-018-1252-3

**Published:** 2018-09-18

**Authors:** Fengxue Xin, Wei Yan, Jie Zhou, Hao Wu, Weiliang Dong, Jiangfeng Ma, Wenming Zhang, Min Jiang

**Affiliations:** 1 State Key Laboratory of Materials-Oriented Chemical Engineering, College of Biotechnology and Pharmaceutical Engineering, Nanjing Tech University, Puzhu South Road 30#, Nanjing, 211816 People’s Republic of China; 2 Jiangsu National Synergetic Innovation Center for Advanced Materials (SICAM), Nanjing Tech University, Nanjing, 211816 People’s Republic of China

**Keywords:** Solventogenic *Clostridium* sp., Fermentation pattern, Substrate utilization, Novel genus

## Abstract

Butanol has been regarded as an important bulk chemical and advanced biofuel; however, large scaling butanol production by solventogenic *Clostridium* sp. is still not economically feasible due to the high cost of substrates, low butanol titer and yield caused by the toxicity of butanol and formation of by-products. Renewed interests in biobutanol as biofuel and rapid development in genetic tools have spurred technological advances to strain modifications. Comprehensive reviews regarding these aspects have been reported elsewhere in detail. Meanwhile, more wild type butanol producers with unique properties were also isolated and characterized. However, few reviews addressed these discoveries of novel wild type solventogenic *Clostridium* sp. strains. Accordingly, this review aims to comprehensively summarize the most recent advances on wild type butanol producers in terms of fermentation patterns, substrate utilization et al. Future perspectives using these native ones as chassis for genetic modification were also discussed.

## Background

Biobutanol has gained great attention as a liquid transportation fuel owning to its similar physical and chemical properties to gasoline in addition to being renewable [1, 2]. Its higher energy content, lower affinity to water, better blending capacities and compatibility with current car engineering make butanol more desirable than ethanol. Biobutanol production through traditional acetone–butanol–ethanol (ABE) fermentation using solventogenic *Clostridium* sp. strains is an industrially established process, and large scaling biobutanol production has been applied for more than 100 years [3–5]. However, it is still not economically feasible due to several obstacles, such as the high cost of traditional feedstocks (starchy based materials), low butanol titer caused by the lipophilic property of butanol and low butanol yield caused by the formation of side products, mainly acetone [6–8].

In nature, solventogenic clostridia, such as *C. acetobutylicum*, *C. beijerinckii* and *C. pasteurianum* are several microorganisms able to ferment monosaccharides, including hexose (e.g., glucose) and pentose (e.g., xylose) to butanol, which typically produce butanol, acetone, and ethanol with a mass ratio of 6:3:1 [6]. However, solventogenic clostridia can not directly utilize polysaccharides, such as cellulose and hemicellulose and other organic wastes, such as glycerol [9]. *C. acetobutylicum* ATCC 824 and *C. beijerinckii* NCIMB 8052 are two most well-known butanol-producing species. Genetic modification of these strains has been comprehensively investigated to improve their fermentation efficiencies, including the improvement of butanol tolerance, simultaneous utilization of glucose and xylose, elimination of acetone production and conversion of acetone into isopropanol [10–13]. However, it should be noticed that due to the complex of ABE fermentation pathway, metabolic re-construction of central metabolic pathway would cause some undesired behaviors. For instance, the disruption of acetoacetate decarboxylase gene (*adc*) in *C. acetobutylicum* could minimize the acetone formation; however, butanol production of the recombinant strain was also affected significantly. Only through the supplementation of costly artificial electron carriers and buffering agency, butanol production was recovered, which makes the butanol production process less economically feasible [13].

Solventogenic clostridia are both spore forming and obligate anaerobes, which have relatively simple growth requirements, and various isolation methods have been well documented [14]. These bacteria have been found to be most commonly associated with living plant materials rather than decaying plant materials or soils. The most recent advances on improvement of butanol production through strain genetic modification and separation technologies have been reviewed in detail elsewhere [2–4, 6]. However, few reviews regarding butanol production using wild type solventogenic strains were reported. As known, native microbes with unique properties such as indigenous elimination of acetone formation could be good chassis for further genetic modification [15, 16]. Hence, in this review, we specifically focused on current advances on butanol production using novel wild type solventogenic *Clostridium* sp. Further perspectives regarding these native butanol producers were also discussed.

## Solventogenic *Clostridium* sp. with untraditional butanol fermentation pattern

In ABE fermentation process, acetone generally accounts for 30% of total solvent mass, which will lower the butanol yield. In addition, acetone does not qualify as fuel and should be separated from the final products, which will further increase the following separation cost [3, 17]. Aiming to solve this obstacle, series of work have been carried out to eliminate the acetone formation through genetic modification. The most direct method is to knock out genes related with acetone production. Indeed, the minimization or suppression of acetone production through interruption of acetone synthetic pathway in vivo led to less acetone production; however, butanol production was also significantly affected with the accumulation of more volatile fatty acids (VFAs), mainly acetate and butyrate rather than solvents [18]. For example, the disruption of acetoacetate decarboxylase gene (*adc*) in *C. acetobutylicum* reduced acetone production to negligible levels with only 0.21 g/L; however, only 7.41 g/L of butanol production occurred, which is much lower than that of parent strain (13.62 g/L). The final butanol titer could only reach the similar level through the metabolic regulation of electron flows with the supplementation of artificial electron carriers, methyl viologen. These artificial electron carriers could alter the carbon flux towards solvents rather than VFAs formation, and butanol production capability could then be recovered [19]. However, this will make the recombinant strain less competitive due to the raised cost caused by the supplementation of expensive electron carriers. On the contrary, indigenous acetone-uncoupled solventogenic *Clostridium* sp. shows promising alternative for butanol production. For instance, Gong et al. found that native *C. tetanomorphum* strain DSM665 could re-assimilate butyrate and acetate for butanol–ethanol (BE) production with the indigenous elimination of acetone. After the process optimization, strain DSM665 could produce 9.81 g/L of butanol and 1.01 g/L of ethanol. Further enzymatic analysis indicated that the activity of acetate/butyrate: acetoacetyl-CoA transferase responsible for acetone production was not detected in *C. tetanomorphum* strain DSM665 [20]. The genome sequence analysis showed that no genes of *ctfA/B* and* adc* exist, which are typically parts of an acetone synthesis pathway in *C. tetanomorphum*. Taken together, compared to the strategy of acetone elimination by metabolic engineering, adoption of native acetone uncoupled solventogenic *Clostridium* strains will not affect butanol production, which could be used as the robust chassis to further improve the butanol titer.

**Fig. 1:**
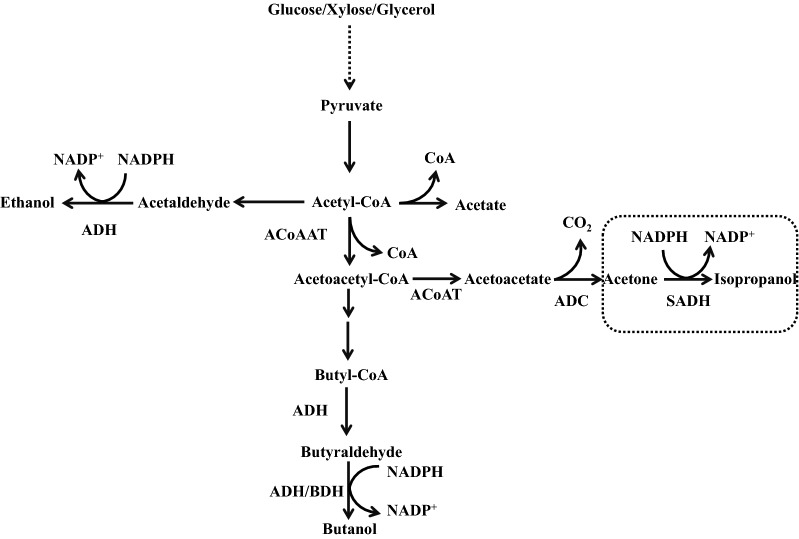
The metabolic pathway for isopropanol–butanol–ethanol (IBE) production. The box shows the synthetic pathway for isopropanol production from acetone. *ACoAAT* acetyl-CoA acetyltransferase, *ACoAT* acetoacetyl-CoA transferase, *ADC* acetoacetate decarboxylase, *SADH* secondary alcohol dehydrogenase, *ADH* alcohol dehydrogenase, *BDH* butanol dehydrogenase

Alternatively, further conversion of acetone into more value-added product, such as isopropanol, offers another promising method [21]. In addition to the introduction of isopropanol synthesizing genes into solventogenic *Clostridium* sp., some native solventogenic *Clostridium* sp. also show the capability of direct isopropanol production. *C. beijerinckii* NRRL B-593 was the first identified one to produce isopropanol–butanol–ethanol (IBE) through the indigenous expression of s-alcohol dehydrogenase (*s-ADH*), which has been comprehensively adopted as the genetic tool for conversion of acetone into isopropanol by other solventogenic *Clostridium* sp., such as *C. acetobutylicum* [22]. However, isopropanol production levels are not conclusive based on different studies. Until now, more isopropanol producing organisms have been isolated and characterized. For instance, Ng et al. [23] isolated *Clostridium* sp. BT10-6, M10-1 and PU31-4 from the nature reserve soil. Especially, strain BT10-6 could accumulate up to 5.26 g/L of isopropanol after 48 h with isopropanol productivity of 0.13 g/L/h, which is 4.6 times of 0.029 g/L/h given by *C. beijerinckii* NRRL B593. Meanwhile, BT10-6 could produce a slightly lower butanol titer and higher butanol productivity compared to *C. beijerinckii* NCIMB 8052. However, the detailed mechanism was not addressed. More recently, Xin et al. [24] isolated another native isopropanol–butanol (IB) producer, *Clostridium* sp. strain NJP7, which also shows efficient solvent production from polysaccharides, such as hemicellulose through consolidated bioprocessing (CBP). With the enhancement of buffering capacity and alcohol dehydrogenase activities through the supplementation of NADPH precursor—nicotinamide, butanol and isopropanol titer by *Clostridium* sp. strain NJP7 was finally improved to 12.21 g/L and 1.92 g/L, respectively. Solvent productivity could also be enhanced to 0.44 g/L/h. Furthermore, with in situ extraction using biodiesel, the amount of butanol and isopropanol was finally improved to 25.58 g/L and 5.25 g/L in the fed batch mode. Meanwhile, some other native IB producers were also discovered, such as *Clostridium* sp. strain A1424, whose 16S rRNA sequence shows 99.92% similarity to *C. diolis* DSM 5431. Strain A1424 could produce 9.43 g/L of butanol and 4.49 g/L of isopropanol at pH 5.5 with the consumption of 46.35 g/L of glucose, respectively, which are the highest values in glucose-based batch fermentations using natural IB producers [11]. It is known that sADH is generally NADPH dependent; hence, NADPH regulation with the supplementation of various electron carriers could be an efficient way for further improvement of isopropanol production [25] (Fig. 1).

## Solventogenic *Clostridium* sp. with expanded spectra of substrate utilization

### Solventogenic *Clostridium* sp. with capability of simultaneous utilization of glucose and xylose in lignocellulosic hydrolysate

The efficient and simultaneous conversion of both pentose and hexose is a significant hurdle to the economic utilization of ligno-biomass hydrolysates for the second biofuel generation [26, 27]. Scientists noted “the lack of a microorganism able to efficiently ferment all sugars released by the hydrolysis from lignocellulosic materials has been one of main factors preventing utilization of lignocellulose”, because either the desired microorganism is unable to utilize pentose at all (e.g., *Saccharomyces cerevisiae*) or the organism consumes the sugar mixture sequentially (e.g., first glucose and then xylose) [6, 28]. Unless both glucose and xylose are utilized, the economic of converting lignocellulosic biomass into bio-based products is unfavorable. Solventogenic clostridia, such as *C. acetobutylicum*, *C. beijerinckii* and *C. pasteurianum* are several organisms able to ferment both pentose and hexose sugars, while high butanol and solvent production and good solvent yields are still obtained from hexose, mainly glucose, whereas significantly lower values are found with pentose, such as xylose [29, 30]. *C. acetobutylicum* ATCC 824 and *C. beijerinckii* NCIMB 8052 have shown this inherent xylose metabolism bottleneck. For example, compared to 11.25 g/L of butanol production with 100% utilization of glucose in P2 medium containing 60 g/L of glucose by *C. acetobutylicum* ATCC 824, only 4.23 g/L of butanol was produced with the residue of 36.73 g/L of xylose under paralleled conditions [30]. When grown in XHP2 medium using 30 g/L of xylose as the sole carbon source, *C. beijerinckii* NCIMB 8052 could only consume 20.20 g/L of xylose [31]. The inefficient xylose utilization leads to another big issue that utilization of xylose is severely inhibited when a preferred carbon source, such as glucose exists in the glucose and xylose mixtures [32]. This phenomenon is referred as carbon catabolite repression (CCR), in which microorganisms preferentially utilize a rapidly metabolizable carbon source, along with the inhibition of some genes expression and enzyme activities related to the catabolism of non-preferred carbon resources [33].

**Fig. 2:**
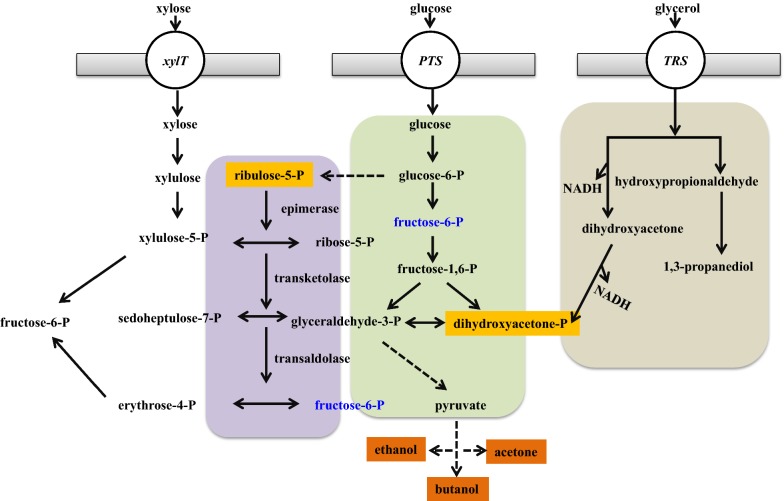
The metabolic pathway for acetone**–**butanol**–**ethanol (ABE) production from glucose, xylose and glycerol

A novel solventogenic *Clostridium* sp. strain BOH3 has been screened, which showed the highest sugar utilization (100%) and butanol production (14.94 g/L) capability compared to other reported wild and mutant strains. More importantly, it could produce similar amount of butanol from xylose to glucose [34]. In solventogenic *Clostridium* species, the non-oxidative pentose phosphate pathway (PPP) is really the only way, by which pentose can be introduced into Embden–Meyerhof–Parnas (EMP) pathway with the final conversion of pyruvate, ATP and NADH [35, 36]. EMP is the central carbon metabolism for hexose sugars utilization, while PPP (also called hexose monophosphate shunt), which is a major source of pentose phosphates for the supply of C5 and C4 units for biosynthesis and reducing power, is another branch of hexose metabolism [37]. As indicated by epimerase–transketolase–transaldolase mediating PPP detection and radiotracer studies, equivalent quantities of pyruvate, ATP and NADH are formed per gram of xylose or glucose fermented [38, 39]. Under the condition of the same amount of sugar utilization, these explain why the products and cell yields of *Clostridium* sp. strain BOH3 on glucose and on xylose are nearly identical (Fig. 2).

**Table 1:** Solvent production by different native *Clostridium* sp. strains

Strain	Substrate	Fermentation pattern	Solvents (g/L)	References
*Clostridium* sp. strain BOH3	Xylose	Acetone–butanol–ethanol (ABE)	5.32 g/L acetone, 14.94 g/L butanol, 1.25 g/L ethanol	[34]
*C. tetanomorphum* strain DSM665	Glucose	Butanol–ethanol (BE)	9.81 g/L butanol, 1.01 g/L ethanol	[20]
*C. pasteurianum* DSM 525	Glycerol	Butanol–propanediol	7.13 g/L butanol, 6.79 g/L 1,3-propanediol	[44]
*Clostridium* sp. BT10-6	Glucose	Isopropanol	5.26 g/L isopropanol	[23]
*Clostridium* sp. strain NJP7	Glucose	Isopropanol–butanol (IB)	12.21 g/L butanol, 1.92 g/L ethanol	[24]
*C. pasteurianum* strain GL11	Glycerol	Butanol–ethanol (BE)	14.7 g/L butanol	[46]

### Efficient metabolism of glycerol to butanol

In addition to lignocellulosic materials, some other organic wastes are also ideal substrates for biobutanol production, such as waste glycerol. As known, glycerol is a cheaper, simpler and more abundant molecule than glucose, which can be taken up into microbial cells through the facilitated diffusion [40]. Many microorganisms have been reported to possess metabolic pathways that can convert glycerol into different metabolic products, including butanol, 1,3-propanediol et al. However, the most studied solventogenic organisms of *C. acetobutylicum* and *C. beijerinckii* are unable to grow solely on glycerol, as they cannot re-oxidize the excess NADH generated in the cellular glycerol catabolism. *C. pasteurianum*, which was first described in 1983 could indigenously metabolize glycerol for butanol synthesis, representing the best studied organism able to naturally produce butanol with glycerol as the sole carbon and energy source. The main product of this fermentation type was butanol; however, 1,3-propanediol and ethanol were also produced [41, 42] (Fig. 2). It should be mentioned that when *C. pasteurianum* is fermented with glucose, it produces mostly VFAs rather than solvents, and glycerol has been proved as the best substrate for butanol production by *C. pasteurianum*. Butanol formation alone by *C. pasteurianum* has been shown to be energetically preferred and its pathway has a neutral redox balance. However, along with the formation of biomass, it is suggested that 1,3-propanediol has to be produced to balance reducing equivalents [43].

Currently, *C. pasteurianum* DSM 525 was the most commonly used one for butanol production from glycerol. 7.13 g/L of butanol and 6.79 g/L of 1,3-propanediol could be produced from 46.38 g/L of glycerol by *C. pasteurianum* DSM 525 [44]. After fermentation process optimization, the final butanol and 1,3-propanediol titers could be further improved to 13.92 g/L and 5.34 g/L, respectively [45]. However, the high ratio of 1,3-propanediol to butanol leads to low butanol yield and also causes the increased cost associated with downstream product recovery. Recently, solventogenic and glycerol utilizing *C. pasteurianum* strain GL11 was successfully isolated, which could produce 14.72 g/L of butanol from 34 g/L of glycerol without formation of by-products including acetone and 1,3-propanediol (1,3-PDO). The elimination of byproducts gave a high butanol yield of 0.43 g/g from glycerol. With further in situ extraction using biodiesel, the butanol production was finally improved to 28.83 g/L in the fed batch mode. Further genomic and enzymatic analysis showed that the deficiency of key enzymes involved in acetone and 1,3-PDO pathways within strain GL11 led to the elimination of these by-products, which also may greatly simplify the downstream separation. Both the elimination of acetone and 1,3-PDO and high butanol tolerance contributed to its high butanol production yield from glycerol, surpassing the theoretical yield from glucose [46]. Thus, strain GL11 could be a very promising chassis for further improvement of butanol production and shows great potential for large scaling production of butanol from waste glycerol (Table 1).

### Butanol producers capable of utilizing gaseous substrates

Syngas, a mixture of hydrogen (H_2_), carbon monoxide (CO) and carbon dioxide (CO_2_) can be used as carbon and energy sources by several solventogenic microorganisms. Butanol production from syngas is only scarcely found in some autotrophic acetogens. During these, solventogenic *C. carboxidivorans* has been reported to possess the ability to convert syngas to butanol [14, 47]. However, it should be noticed that butanol fermentation pattern did not follow the traditional ABE one, as acetone was normally not found in these species, while other solvents like hexanol could be formed. As far as known, the C4 formation pathway is identical to that in *C. acetobutylicum*. A thiolase (ThlA) combines two molecules of acetyl-CoA into acetoacetyl-CoA, which in turn is reduced to 3-hydroxybutyryl-CoA by 3-hydroxybutyryl-CoA dehydrogenase (Hbd). Then, crotonase (Crt) dehydrates 3-hydroxybutyryl-CoA to crotonyl-CoA, which is finally reduced to butyryl-CoA by electron-bifurcating butyryl-CoA dehydrogenase (Bcd) complex [48]. Butyryl-CoA can then either be converted into butyrate (by phosphotransbutyrylase and butyrate kinase or by acetate:coenzyme A transferase and acetate kinase, respectively) or to butanol (by butyraldehyde and butanol dehydrogenases). Coenzyme specificity has been determined for only few butyraldehyde and butanol dehydrogenases. The bifunctional enzyme adhE2 from *C. acetobutylicum* uses NADH for both reactions and has been used in construction of autotrophic recombinants [49].

*C. carboxidivorans* turned out to be the species that scientific attention focused on recently, which is also able to form the C6 compound of hexanol. It was found that low partial pressure of CO in the headspace, coupled with restricted mass transfer for CO and H_2,_ was required for the successful fermentation of *C. carboxidivorans* strain P7. In the absence of substrate inhibition (particularly from CO), growth limitation increased alcohols production, especially butanol and hexanol. 1.00 g/L of butanol, 1.00 g/L of hexanol and 3.00 g/L of ethanol were achieved in bottle fermentations. Different from sugar-based medium, operating conditions significantly affect the metabolic fermentation profiles and butanol accumulation of *C. carb.oxidivorans*. In batch fermentation without pH regulation, acetic acid, butyric acid and ethanol were detected while only negligible butanol production was observed after complete substrate exhaustion. Maximum ethanol and butanol concentrations in the bioreactors were obtained at pH 5.75, reaching values of 5.55 and 2.66 g/L, respectively, demonstrating that low pH was more favorable to solventogenesis in this process, although it negatively affects biomass growth, which also plays a role in the final alcohol titer [47]. Currently, the highest butanol concentration obtained with the natural butanol producer *C. carboxidivorans* is still much lower than that obtained with mutants of *C. acetobutylicum* [14]. Thus, it is tempting to try a metabolic engineering approach to enhance butanol production capacity.

## Novel bacterial genus for butanol production

Extensive works regarding genetical construction of some model microorganisms, such as *Escherichia coli* and *S. cerevisiae,* have been carried out for the achievement of biobutanol production [50–53]. Not only limited to expanding butanol producers through metabolic engineering, some other bacterial genus with the capability of butanol production was also isolated and characterized. Recently, our group has isolated and characterized a new thermophilic *Thermoanaerobacterium* sp. M5, which could directly produce butanol from hemicellulose through CBP at 55 °C, which features hydrolytic enzymes production, enzymatic hydrolysis and butanol fermentation in one step. Moreover, strain M5 possesses a unique BE pathway with the elimination of acetone [54]. Generally, thermophilic conditions could avoid microbial contamination, decrease the cooling system cost and further facilitate the downstream product recovery. Currently, most wild type thermophilic strains, such as *C. thermocellum*, *T. saccharolyticum*, *Geobacillus thermoglucosidasius* et al. have been reported to produce ethanol alone [17]. This newly isolated and characterized butanologenic strain will broaden our knowledge and add to the pool of known butanol generating microbes.

Through the optimization of fermentation process, strain M5 could produce 1.17 g/L of butanol from xylan to CBP, in which xylanase production and butanol fermentation were both completed by strain M5. Genomic and proteomic analysis showed that the capabilities of efficient hemicellulose degradation and butanol synthesis were attributed to the efficient expression of hemicellulose degradation systems, mainly xylanase and β-xylosidase, and the bifunctional alcohol/aldehyde dehydrogenase (AdhE). Owning to its efficient hemicellulose degradation systems, the co-cultivation system consisting of *Thermoanaerobacterium* sp. M5 and solventogenic *C. acetobutylicum* NJ4 could further improve the final butanol titer to 8.34 g/L, representing the highest butanol production from xylan to CBP. It should be noticed that high amount of butyric acid was formed compared to solvents; hence, further genetic overexpression of AdhE may help to improve the final butanol titer. As a newly identified thermophilic and butanologenic strain, more detailed analysis of enzymes responsible for butanol formation is still needed, and more thermophilic enzymes could be identified and adopted as genetic tools for other strains’ modification [55].

## Conclusions and future perspectives

Large scaling biobutanol production through ABE fermentation process remains a great challenge, primarily due to the low butanol titer caused by the toxicity of butanol to cells and low yield caused by the formation of by products, mainly acetone [56]. Over the past few years, strategies for achieving high butanol production with low acetone production by solventogenic *Clostridium* sp. have been well developed. Currently, acetone formation could be completely suppressed or converted into more valuable product, such as isopropanol through metabolic engineering or newly isolated strains. In parallel, the rapid development of genetic tools for molecular characterization of complex phenotypes has led to numerous new insights into butanol tolerance, and various solventogenic *Clostridium* sp. have been genetically engineered to improve the butanol tolerance and final butanol titer. However, due to the complex of metabolic regulation of proteins or enzymes involved in the butanol tolerance, there is still lack of sufficient tools to significantly improve the final butanol titer. It has been proved that the most efficient method is still through the traditional chemical mutagenesis; however, butanol production still maintained at low levels (below 20 g/L), which can not compete with ethanol production. Integration of novel strain isolation and metabolic evolution may pave a way for selection of more promising butanol producers [57–61].
